# Efficacy and Safety of a Single-Dose Mebendazole 500 mg Chewable, Rapidly-Disintegrating Tablet for *Ascaris lumbricoides* and *Trichuris trichiura* Infection Treatment in Pediatric Patients: A Double-Blind, Randomized, Placebo-Controlled, Phase 3 Study

**DOI:** 10.4269/ajtmh.17-0108

**Published:** 2017-09-05

**Authors:** Steven A. Silber, Ermias Diro, Netsanet Workneh, Zeleke Mekonnen, Bruno Levecke, Peter Steinmann, Irenee Umulisa, Hailemaryam Alemu, Benny Baeten, Marc Engelen, Peter Hu, Andrew Friedman, Alan Baseman, Joseph Mrus

**Affiliations:** 1 Janssen Research & Development LLC, Raritan, New Jersey;; 2 University of Gondar, Gondar, Ethiopia;; 3 Department of Pediatrics, College of Public Health and Medical Sciences, Jimma University, Jimma, Ethiopia;; 4 Department of Medical Laboratory Sciences and Pathology, College of Public Health and Medical Sciences, Jimma University, Jimma, Ethiopia;; 5 Department of Virology, Parasitology and Immunology, Ghent University, Merelbeke, Belgium;; 6 Swiss Tropical and Public Health Institute, Basel, Switzerland;; 7 University of Basel, Basel, Switzerland;; 8 Rwanda Biomedical Centre, Kigali, Rwanda;; 9 Janssen Pharmaceutica NV, Beerse, Belgium

## Abstract

This randomized, double-blind, placebo-controlled study evaluated the efficacy and safety of a new chewable, rapidly-disintegrating mebendazole (MBZ) 500 mg tablet for *Ascaris lumbricoides* and *Trichuris trichiura* infection treatment. Pediatric patients (1–15 years; *N* = 295; from Ethiopia and Rwanda) excreting *A. lumbricoides* and/or *T. trichiura* eggs were enrolled. The study had a screening phase (3 days), a double-blind treatment phase (DBP, 19 days), and an open-label phase (OLP, 7 days). Patients received MBZ or placebo on day 1 of DBP and open-label MBZ on day 19 ± 2 after stool sample collection. Cure rates (primary endpoint), defined as species-specific egg count of 0 at the end of DBP, were significantly higher in the MBZ group than placebo for *A. lumbricoides* (83.7% [72/86; 95% CI: 74.2%; 90.8%] versus 11.1% [9/81; 95% CI: 5.2%; 20.1%], *P* < 0.001) and for *T. trichiura* (33.9% [42/124; 95% CI: 25.6%; 42.9%] versus 7.6% [9/119; 95% CI: 3.5%; 13.9%], *P* < 0.001). Egg reduction rates (secondary endpoint) were significantly higher in the MBZ group than placebo for *A. lumbricoides* (97.9% [95% CI: 94.4; 99.9] versus 19.2% [95% CI: −5.9; 41.5]; *P* < 0.001) and *T. trichiura* (59.7% [95% CI: 33.9; 78.8] versus 10.5% [95% CI: −16.8; 32.9]; *P* = 0.003). Treatment-emergent adverse events (TEAEs) in MBZ group occurred in 6.3% (9/144) of patients during DBP and 2.5% (7/278) during OLP. No deaths, serious TEAEs, or TEAEs leading to discontinuations were reported. A 500 mg chewable MBZ tablet was more efficacious than placebo for the treatment of *A. lumbricoides* and *T. trichiura* infections in pediatric patients, and no safety concerns were identified.

## INTRODUCTION

Soil-transmitted helminth (STH) infections occur in humans either after inadvertent ingestion of *Ascaris lumbricoides* (roundworm) or *Trichuris trichiura* (whipworm) eggs or *Ancylostoma duodenale* (hookworm) larvae, or after skin penetration by infective larvae in the soil of both hookworm species: *A. duodenale* and *Necator americanus*.^1^ Globally, STHs infect more than 1.5 billion people, with the highest prevalence in sub-Saharan Africa, the Americas, China, and East Asia.^1,2^ *A. lumbrocoides* infections are responsible for 1 × 10^6^ and *T. trichiura* infections for 0.5 × 10^6^ disability adjusted life years,^2^ thus accounting for the highest disease burden among all neglected tropical diseases.^3^

A vast majority of STH infections are seen in children residing in communities lacking adequate sanitary facilities, provision of safe potable water, and basic hygiene practices.^4^ According to the World Health Organization (WHO) estimate in 2015, more than 270 million preschool and 600 million school-aged children are infected or live in areas where there is an increased risk of STH infections.^5,6^ STH infections potentially impact a child’s nutritional status and pose a significant threat to their cognitive and physical development,^7,8^ consequently, resulting in poor school performance and absenteeism limiting educational advancement and economic development.^7,9^

An effective and safe mebendazole (MBZ) 500 mg solid oral tablet formulation is available for the treatment of STH in children older than one year.^10^ The WHO recommends that anthelmintic tablets be crushed and mixed with water for easy administration in children < 3 years of age. Alternatively, a chewable tablet may be administered.^11^ Also, practical limitations, such as bitter taste of crushed medication and risk of choking, necessitate the availability of age-appropriate formulations for children in the age group of 1–5 years.^11^

To address these recommendations, a chewable strawberry-flavored MBZ 500 mg tablet was initially developed as part of the drug development program for young children.^12^ Later, a rapidly-disintegrating formulation, which absorbs water in less than a minute to convert to a semi-solid mass was developed to further improve ease of administration. The primary objective of this study was to demonstrate the efficacy and safety of a single dose of this rapidly-disintegrating chewable MBZ 500 mg tablet formulation compared with placebo in the treatment of *A. lumbricoides* and *T. trichiura* infections in pediatric patients (1–16 years).

## METHODS

The study protocol was approved by the US Food and Drug Administration, the Ethiopian National Research Ethics Review Committees, the Food, Medicine and Health Administration and Control Authority (Ethiopian Regulatory Authority) as well as the Institutional Review Boards of the University of Gondar and Jimma University in Ethiopia. The protocol was also approved by the Rwandan National Ethics Committee, an independent committee under the Rwanda Ministry of Health, Kigali, Rwanda. The study was conducted in accordance with the ethical principles originating in the Declaration of Helsinki, consistent with the International Council for Harmonization Good Clinical Practice guidelines, applicable regulatory requirements, and in compliance with the protocol. The parents or legally accepted guardians of all children provided an informed consent before participation in the study. In addition, children ≥ 6 years were informed about the study and their assent was documented.

### Study design, randomization, and blinding.

**Figure 1.**
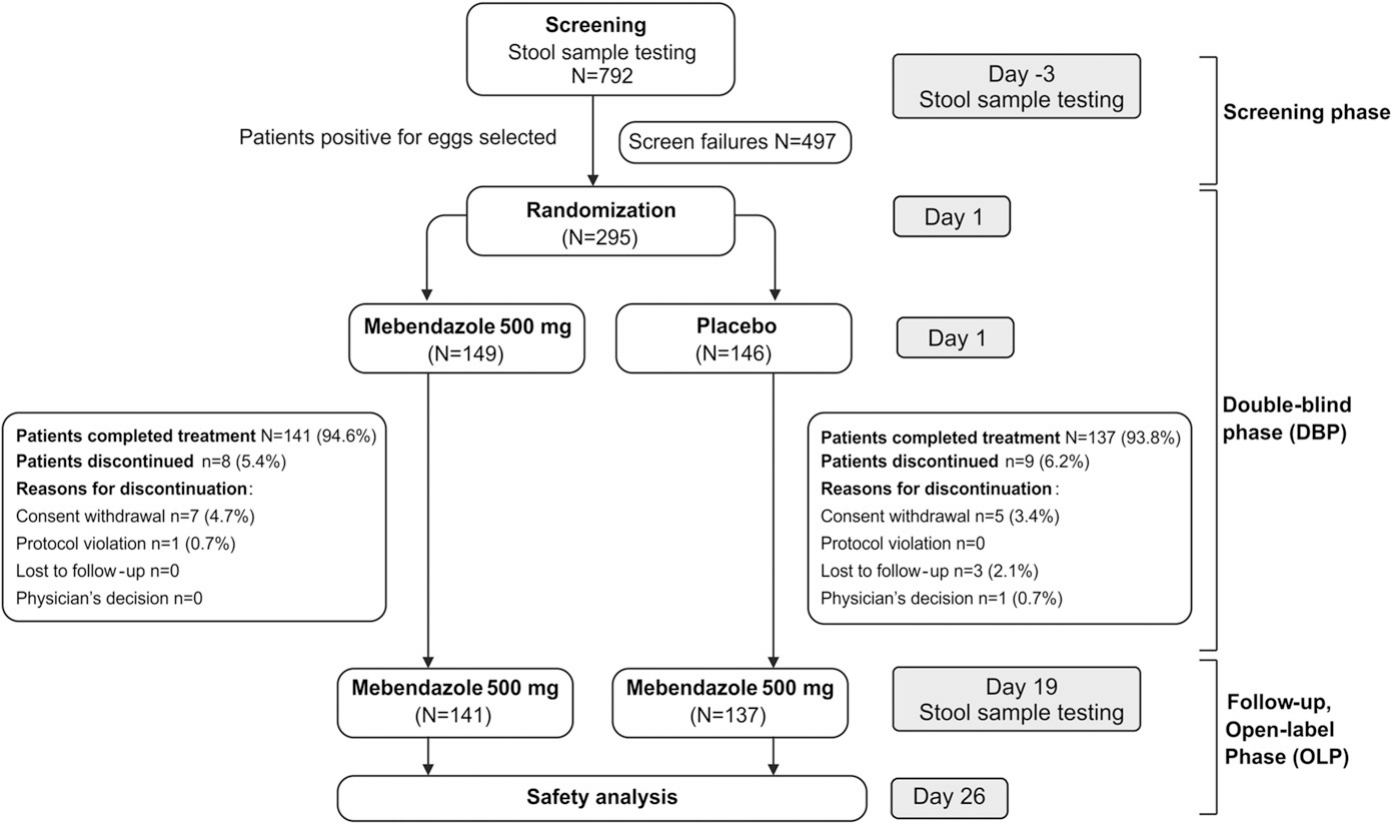
Study design and patient disposition.

This was a phase 3, randomized, double-blind, placebo-controlled study conducted at two sites in Ethiopia ( the University of Gondar and University of Jimma) and one site in Rwanda ( the Rwaza Health Center, Northern Rwanda) between December 8, 2014 and September 3, 2015. The study consisted of screening (3 days), a double-blind treatment phase (DBP, 19 ± 2 days) and follow-up, and an open-label phase (OLP, 7 days) (Figure 1). Post screening, children with *A. lumbricoides* and/or *T. trichiura* eggs in their stools were randomized (1:1) using computer-generated random permuted blocks to receive either a single 500 mg chewable MBZ tablet or placebo. The randomization was implemented using an interactive voice response system. Patients, laboratory personnel, and clinicians were blinded to the randomization. On day 1 of the DBP, the study staff administered the MBZ or placebo tablet to the randomized patients. For children 1 year to < 3 years of age, the tablet was placed in a teaspoon and bottled potable drinking water was poured into the remaining volume of the teaspoon (approximately 2–3 mL). The tablet quickly absorbed the water, converting into a soft semisolid mass, which was then easily administered to the patient. Patients older than 3 years of age chewed the tablet without mixing with water. On day 19, after providing the follow-up stool samples, all randomized patients received a MBZ 500 mg chewable tablet. In the follow-up phase, all patients were evaluated for safety parameters on day 26.

### Study population.

Boys and girls (1–16 years of age) living in high STH-prevalence areas in Ethiopia or Rwanda were screened for STH eggs in stools. Children with *A. lumbricoides* and/or *T. trichiura* eggs in their stools were eligible for randomization. Children positive for hookworm along with one or both of the previously mentioned STH species were also eligible. Children had to be otherwise healthy based on medical history, physical examination, vital signs, hemoglobin, and concomitant medications for inclusion. Exclusion criteria were as follows: children with a history or a medical disorder causing difficulty in chewing or swallowing, significant anemia (< 8 g/dL), active diarrhea (passage of ≥ 3 loose or liquid stools per day), significant wasting (moderate and severe-below minus two standard deviations from median weight for height of reference population), or hypersensitivity to MBZ or any inert ingredients in the chewable formulation or other medications in the benzimidazole class, and girls ≥ 9 years of age having a positive urine pregnancy test at screening or randomization. In addition, children who had taken MBZ or any other treatment of STH infection, who had received an investigational drug (including vaccines) or used an investigational medical device, or who had any preplanned surgery procedures within 30 days before entering into the study were also excluded.

### Efficacy assessments.

A total of two stool samples (days 1 and 19 [±2]) were obtained from each patient. Two Kato-Katz smears were prepared from each of the stool samples to reduce the number of false negative test results, and hence enhance the accuracy of the efficacy estimates.^13,14^ The average egg counts for *A. lumbricoides* or *T. trichiura* from the stool sample collected in pretreatment (day 1) were used as the baseline values, and the average egg counts from the posttreatment (day 19) stool sample were used as the endpoint values for the statistical analysis.

#### Primary endpoint.

The primary efficacy endpoint was the cure rate for *A. lumbricoides* and *T. trichiura* at the end of the double-blind (DB) treatment period (day 19). The cure rate was calculated as the percentage of randomized patients with a zero egg count for *A. lumbricoides* or *T. trichiura* (no eggs present in either smear) posttreatment.

#### Secondary endpoint.

The secondary efficacy endpoint was the egg count reduction rate (ERR) for *A. lumbricoides* and *T. trichiura* at the end of the DB treatment period. The ERR was calculated by evaluating the percentage change in the group arithmetic mean egg count posttreatment compared with baseline for each group.^15,16^

#### Exploratory endpoints.

Exploratory efficacy endpoints included the cure rate and ERR at the end of the DB treatment period for hookworm.

### Safety and tolerability assessments.

Safety assessments included evaluation of vital signs, physical examinations, treatment-emergent adverse events (TEAEs), and serious AEs. The TEAEs were assessed based on the direct observations of the investigator, the patient, or as reported by the patient, parent, or guardian. The TEAEs were assessed separately for the DBP and the OLP. Ease of administration of drug and tolerability were also reported.

### Statistical analysis.

#### Sample size.

A total of 50 patients with *A. lumbricoides* and 200 with *T. trichiura* were targeted to be enrolled in the study. This was required to detect a difference in cure rate between MBZ and placebo of 60% for *A. lumbricoides* and 20% for *T. trichiura* with a power of 90% at the overall two-sided type I error rate of 0.05 based on Fisher’s exact test. This calculation was based on the ranges for cure-rates reported in previous studies.^17^

#### Null hypotheses.

Two null hypotheses were tested sequentially. The first null hypothesis was that the cure rate of *A. lumbricoides* after treatment with a single dose of a 500 mg chewable tablet of MBZ or placebo was not different. The second null hypothesis was that the cure rate of *T. trichiura* after treatment with a single dose of a 500 mg chewable tablet of MBZ or placebo was not different. The second hypothesis was to be evaluated only if the first null hypothesis was rejected at the *P* = 0.05 level.

#### Efficacy and safety analysis.

The primary efficacy analysis was based on the intent-to-treat (ITT) population defined as all randomized patients with a positive pretreatment stool sample. Efficacy for the two infections was calculated separately, and patients with both infections were included in the analysis of both. For the primary efficacy endpoints, the Cochran–Mantel–Haenszel test controlling the effect of site was used to compare the cure rates between the two treatment groups. Patients missing the posttreatment stool sample were considered not cured.

For the secondary efficacy endpoints, the 95% confidence intervals for the ERR were determined by bootstrap analysis. A permutation test was performed to assess differences in the ERR between MBZ (500 mg) and the placebo arm.

The DBP safety analysis population included all randomized patients who received one dose of study drug at baseline. The OLP safety analysis population included all patients who received the 500 mg MBZ on day 19. Exploratory and safety analyses were summarized descriptively.

## RESULTS

### Patient disposition and characteristics.

Of 792 children screened, 295 were enrolled and randomized of which 278 (94.2%) patients completed the study (MBZ: *n* = 141, placebo: *n* = 137). The most common reason for discontinuation was withdrawal of consent (MBZ: 7 [4.7%], placebo: 5 [3.4%]). None of the patients discontinued the study because of noncompliance or TEAEs (Figure 1). Patients enrolled were up to 15 years of age and the mean (SD) age of the patients was 7.8 (3.18) years (Table 1). There were similar proportions of boys and girls in both treatment groups, as well as within each group. At baseline, 167 patients were infected with *A. lumbricoides* (MBZ: *N* = 86, placebo: *N* = 81) and 243 with *T. trichiura* (MBZ: *n* = 124, placebo: *n* = 119); 115 were coinfected with both, and 13 were infected with hookworm species in addition to *A. lumbricoides* and *T. trichiura.*

**Table 1:** Patient demographics and baseline characteristics (ITT population)

	Mebendazole 500 mg (*n* = 149)	Placebo (*n* = 146)	Total (*n* = 295)
Sex, *n* (%)			
Boys	71 (47.7)	72 (49.3)	143 (48.5)
Girls	78 (52.3)	74 (50.7)	152 (51.5)
Age (years)			
Mean (SD)	7.9 (3.27)	7.7 (3.09)	7.8 (3.18)
Baseline BMI (kg/m^2^)			
Mean (SD)	15.7 (1.7)	15.7 (1.9)	15.7 (1.8)
*A. lumbricoides* egg count, *n* (%)			
No infection	63 (42.3)	65 (44.5)	128 (43.4)
Light (1–4,999 eggs/gm)	34 (22.8)	31 (21.2)	65 (22.0)
Moderate (5,000–49,999 eggs/gm)	45 (30.2)	44 (30.1)	89 (30.2)
Heavy (≥ 50,000 eggs/gm)	7 (4.7)	6 (4.1)	13 (4.4)
*T. trichiura* egg count, *n* (%)			
No Infection	25 (16.8)	27 (18.5)	52 (17.6)
Light (1–999 eggs/gm)	100 (67.1)	102 (69.9)	202 (68.5)
Moderate (1,000–9,999 eggs/gm)	24 (16.1)	17 (11.6)	41 (13.9)
Heavy (≥ 10,000 eggs/gm)	0	0	0
Hook worm egg count, *n* (%)			
No infection	145 (97.3)	137 (93.8)	282 (95.6)
Light (1–1,999 eggs/gm)	4 (2.7)	8 (5.5)	12 (4.1)
Moderate (2,000–3,999 eggs/gm)	0 (0)	1 (0.7)	1 (0.3)
Heavy (≥ 4,000 eggs/gm)	0	0	0

BMI = body mass index; ITT = intent-to-treat.

### Efficacy.

#### Primary endpoint.

Cure rates were significantly higher in patients receiving MBZ versus placebo for *A. lumbricoides* (MBZ: 83.7% [95% CI: 74.2%; 90.8%], placebo: 11.1% [95% CI: 5.2%; 20.1%]; *P* < 0.001) and *T. trichiura* (MBZ: 33.9% [95% CI: 25.6%; 42.9%], placebo: 7.6% [95% CI: 3.5%; 13.9%)]; *P* < 0.001) (Table 2). The cure rate of *A. lumbricoide*s for patients in all age groups (> 3 years, 3–6 years and 7–15 years) was ≥ 80% for the MBZ group. The cure rate of *T. trichiura* for patients in all age groups was > 20% for the MBZ group with the 7–15 years group exhibiting the highest cure rate (37.1%) among all the age groups (Supplemental Table 1). Both null hypotheses that the MBZ and placebo group demonstrated similar efficacy were, therefore, rejected.

**Table 2:** The cure rate and egg reduction rate in pediatric patients infected with *A. lumbricoides* and *T. trichiura* (ITT population)

	*A. lumbricoides*		*T. trichiura*	
	Mebendazole 500 mg (*n* = 86)	Placebo (*n* = 81)	Mebendazole 500 mg (*n* = 124)	Placebo (*n* = 119)
Baseline (eggs/gm)				
*n*	86	81	124	119
Mean (SD)	17,610.1 (23,476.8)	16,959.6 (20,684.0)	647.8 (1,256.2)	584.5 (930.1)
Cure at day 19				
*n* (%)	72 (83.7)	9 (11.1)	42 (33.9)	9 (7.6)
95% CI	(74.2; 90.8)	(5.2; 20.1)	(25.6; 42.9)	(3.5; 13.9)
*P* value	< 0.001		< 0.001	
Egg count at day 19 (eggs/gm)				
*n*	81	76	118	112
Mean (SD)	366.7 (2,325.3)	13,706.7 (23,168.3)	260.7 (1,042.9)	523.0 (1,020.9)
Egg count reduction rate (%)	97.9	19.2	59.7	10.5
*P* value	< 0.001		0.003	
95% CI	(94.4; 99.9)	(−5.9; 41.5)	(33.9; 78.8)	(−16.8; 32.9)

CI = confidence interval; gm = gram; ITT = intent-to-treat; SD = standard deviation.

#### Secondary endpoint.

The ERRs as measured by group arithmetic mean were also significantly different between MBZ and placebo in patients infected with *A. lumbricoides* (MBZ: 97.9%, [95% CI: 94.4; 99.9]; placebo: 19.2%, [95% CI: −5.9; 41.5]; *P* < 0.001) and *T. trichiura* (MBZ: 59.7% [95% CI: 33.9; 78.8]; placebo: 10.5% [95% CI: −16.8; 32.9], *P* = 0.003) (Table 2).

#### Exploratory endpoint.

At baseline, 13 patients were infected with hookworm species (MBZ: *n* = 4, placebo: *n* = 9). The cure rate and ERR for the MBZ group was 100%. For the placebo group, the cure rate was 22.2% and the ERR was 17.5%.

### Safety.

There were no deaths, serious AEs, or TEAEs leading to discontinuation in this study. Overall, the incidence of TEAEs was generally comparable between the groups over the DBP, with similar rates of TEAEs for MBZ (9/144 [6.3%]) and placebo (8/140 [5.7%]); TEAEs were mild or moderate in intensity. The most common TEAEs in both groups included gastrointestinal disorders (MBZ: 3/144 [2.1%], placebo: 2/140 [1.4%]) and infections (MBZ: 3/144 [2.1%], placebo: 4/140 [2.9%]) (Table 3). Abdominal pain (*n* = 1), abdominal distension (*n* = 2), and rash (*n* = 1) were the only TEAEs considered possibly related to MBZ treatment.

**Table 3:** Treatment-emergent adverse events in the double-blind phase (safety analysis set)

	Mebendazole 500 mg (*n* = 144) *n* (%)	Placebo (*n* = 140) *n* (%)
Total no. children with any TEAE	9 (6.3)	8 (5.7)
Gastrointestinal disorders	3 (2.1)	2 (1.4)
Abdominal distension	2 (1.4)	1 (0.7)
Abdominal pain	1 (0.7)	1 (0.7)
Infections and infestations	3 (2.1)	4 (2.9)
Gastroenteritis	1 (0.7)	0
Nasopharyngitis	1 (0.7)	2 (1.4)
Tinea infestation	1 (0.7)	0
Conjunctivitis	0	1 (0.7)
Conjunctivitis bacterial	0	1 (0.7)
Tonsillitis	0	1 (0.7)
Metabolism and nutrition disorders	1 (0.7)	0
Vitamin A deficiency	1 (0.7)	0
Respiratory, thoracic, and mediastinal disorders	1 (0.7)	2 (1.4)
Cough	1 (0.7)	2 (1.4)
Skin and subcutaneous tissue disorders	1 (0.7)	0
Rash pruritic	1 (0.7)	0
Eye disorders	0	1 (0.7)
Night blindness	0	1 (0.7)

TEAE = treatment-emergent adverse event.

The overall incidence of TEAEs in the OLP (day 19–day 26) was 2.5% (7/278 patients), with diarrhea being the most common (2/278; 0.7%). Diarrhea (*n* = 2), abdominal pain (*n* = 1), and vomiting (*n* = 1) were considered possibly related to MBZ. No clinically significant changes were observed in vital signs or physical examination.

### Tolerability.

Of the 284 patients receiving the study drug or placebo in the DBP of the study, 259 (91.2%) chewed the tablets, and 25 (8.8%) patients received the study drug mixed with water in a spoon. There were no instances of choking or vomiting in either treatment group. There were three instances of gagging (MBZ: *n* = 2, placebo: *n* = 1) and two instances of difficulty in swallowing in the placebo group; all instances were in patients who were < 3 years old (Table 4). In the OLP, among the 278 patients, 24 (8.6%) received the study drug mixed with water in a spoon, and the remaining 254 (91.4%) patients chewed the tablets. There were no instances of choking, vomiting, or gagging. In patients < 3 years old, there was one instance of difficulty in swallowing medicine and two instances of consuming more than half the medication and spitting it out.

**Table 4:** Drug administration tolerability profile in the double-blind phase (safety analysis set)

	Mebendazole 500 mg (*n* = 144)	Placebo (*n* = 140)	Total (*n* = 284)
Ease of administration, *n* (%)			
Chewed	131 (91.0)	128 (91.4)	259 (91.2)
Swallowed	0	0	0
Mixed with water and given with spoon	13 (9.0)	12 (8.6)	25 (8.8)
Tolerability, *n* (%)			
Choking	0	0	0
Gagging	2 (1.4)	1 (0.7)	3 (1.1)
Vomiting	0	0	0
Difficulty swallowing	0	2 (1.4)*	2 (0.7)
Spitting out of medication	0	2 (1.4)*	2 (0.7)
Refusal to take the medication	0	2 (1.4)*	2 (0.7)

* The same two patients in the placebo group reported difficulty swallowing, spit out, and refused medication.

## DISCUSSION

The present phase 3, randomized, placebo-controlled, double-blind study demonstrated that a single 500 mg chewable, rapidly-disintegrating tablet of MBZ is effective for the treatment of *A. lumbricoides* and *T. trichiura* infections in pediatric patients as indicated by significantly higher cure rates and ERRs compared with placebo for both STH species. The MBZ treatment demonstrated a consistent safety and tolerability profile to that of earlier studies, with no safety concerns identified.

In 2001, the World Health Assembly resolved to eliminate STH as a childhood health-problem. One of the targets was to regularly administer preventive therapy to at least 75% of school-aged children at high risk of STH infection associated morbidity by 2010. However, this was achieved in only about one-third of the previously mentioned population. In 2011, the WHO revised its strategic plan to eliminate morbidity caused by STH (defined as a prevalence of < 1% moderate to heavy infections) as a health issue in children by 2020. This includes guidance to STH-endemic region governments, partners, and donors with therapy requirements to achieve the goal.^18^

One of the major challenges in achieving this goal is the poor adherence of children to antihelminthics due to tolerability issues and difficulty in administration. To overcome this challenge, the WHO recommends that for children 1 to < 3 years of age, chewable tablets should be crushed and given with a small quantity of water.^11^ Consistent with the WHO recommendations, a rapidly-disintegrating chewable strawberry-flavored 500 mg MBZ tablet was developed. The tablet does not require crushing and converts into a semisolid mass when a small amount of water is added, thereby improving palatability and ease of administration of MBZ in preschool and school-aged children.

The two countries chosen to conduct this study, Ethiopia and Rwanda, have been shown to have a high prevalence of STH infections both among preschool-aged^19^ and school-aged children.^20,21^ The efficacy results of this study are similar to those reported previously in literature.^(16,22–24)^ The cure rate for *A. lumbricoides* (83.7%) and *T. trichiura* (33.9%) was in the range of other studies using 500 mg single-dose MBZ (72.5–100.0% and 8.4–100.0% respectively).^16,24^ In our study, analysis by age group for each helminth species demonstrated similar cure rates across all three age groups. These results were based on a small sample size in younger age subgroups (< 3 and 3–6 years).

Data from previous studies with MBZ 500 mg solid oral tablet dosed once daily for *A. lumbricoides* demonstrated ERRs ranging from 89.8% to 100.0% and 31.6% to 93.0% for *T. trichiura*.^17,25^ The ERR of *A. lumbricoides* (97.9%) and *T. trichiura* (57.9%) reported in the current study fall within the reported range. The cure rates and ERR rates reported with the chewable rapidly-disintegrating formulation are comparable with those observed with the solid oral tablet. This is an important finding given the WHO estimates that approximately 250 million infected or at-risk children are < 5 years of age. Only a small number of patients had heavy *A. lumbricoides* infestation (i.e., ≥ 50,000 eggs/gm of feces; *n* = 13), and no patients had heavy *T. trichiura* infestation (≥ 10,000 eggs/gm). Hence, no conclusions can be drawn on the efficacy and safety of the chewable MBZ tablet in patients with infestations of heavy intensity.

No deaths or serious AEs were observed. The incidence of TEAEs in the present study (6.3%) was lower than in a previous safety study (11%) with an earlier version of MBZ chewable tablet.^13,26^ All TEAEs were considered mild or moderate and most were not considered related to MBZ administration. The present study also demonstrated that MBZ can be used safely in children aged 12–36 months. In this study, the tablet was successfully chewed by all patients who were ≥ 3 years of age. For patients < 3 years of age, mixing with water (without crushing) allowed for easy administration as a semisolid mass to 25 patients. Two patients in the MBZ group reported gagging in DBP and one instance of difficulty in swallowing and two instances of spitting out medication after swallowing in OLP, but there were no instances of choking.

## CONCLUSIONS

The present study demonstrates that a single-dose, chewable MBZ 500 mg tablet is efficacious in the treatment of *A. lumbricoides* and *T. trichiura* infections in pediatric patients between 1 and 15 years of age. This chewable, rapidly-disintegrating formulation has a safety and tolerability profile consistent with previous formulations and could facilitate the treatment of young children who have difficulty swallowing a tablet, thus potentially expanding the population of at-risk children that can be effectively treated in mass drug administration programs for STH infections.

## Supplementary Material

Supplemental Table.
