# Supplementary material for: Efficacy and Safety of a Single-Dose Mebendazole 500 mg Chewable, Rapidly-Disintegrating Tablet for Ascaris lumbricoides and Trichuris trichiura Infection Treatment in Pediatric Patients: A Double-Blind, Randomized, Placebo-Controlled, Phase 3 Study
Source: Am J Trop Med Hyg. 2017 Sep 5;97(6):1851–6. doi: 10.4269/ajtmh.17-0108 (PMC5805036; doi:10.4269/ajtmh.17-0108)
Supplement: Supplementary file 1 [file tpmd170108.SD1.pdf]

Supplemental Table 1

The cure rate in pediatric patients infected with *A. lumbricoides* and *T. trichiura* (ITT population)

	< 3 years		3–6 years		7–15 years	
A. lumbricoides	Mebendazole 500 mg (*n* = 9)	Placebo (*n* = 9)	Mebendazole 500 mg (*n* = 10)	Placebo (*n* = 9)	Mebendazole 500 mg (*n* = 67)	Placebo (*n* = 63)
Baseline (eggs/gm)						
n	9	9	10	9	67	63
Mean (SD)	24,822.7 (37,876.3)	15,222.7 (25,810.4)	21,591.6 (26,370.5)	9,542.7 (9,779.83)	16,047.0 (20,715.5)	18,267.2 (21,061.3)
Egg count at day 19 (eggs/gm)						
n	8	8	9	8	64	60
Mean (SD)	0 (0)	24,558.0 (49,706.8)	2.7 (8.0)	5,011.5 (6,835.3)	463.7 (2,611.6)	13,419.2 (18,903.6)
Cure at day 19						
n (%)	8 (88.9)	2 (22.2)	8 (80.0)	2 (22.2)	56 (83.6)	5 (7.9)

	< 3 years		3–6 years		7–15 years	
T. trichiura	Mebendazole 500 mg (*n* = 6)	Placebo (*n* = 10)	Mebendazole 500 mg (*n* = 29)	Placebo (*n* = 21)	Mebendazole 500 mg (*n* = 89)	Placebo (*n* = 88)
Baseline (eggs/gm)						
n	6	10	29	21	67	63
Mean (SD)	568.0 (838.4)	874.8 (1,788.1)	675.7 (1,660.75)	690.3 (866.1)	644.1 (1,134.7)	584.5 (930.1)
Egg count at day 19 (eggs/gm)						
n	4	9	29	21	85	60
Mean (SD)	210 (265.4)	760.0 (1,204.1)	174.6 (230.6)	461.1 (452.4)	512.8 (1,107.8)	523.0 (1,020.9)
Cure at day 19						
n (%)	2 (33.3)	1 (10.0)	7 (24.1)	1 (4.8)	33 (37.1)	7 (8.0)

CI = confidence interval; gm = gram; ITT = intent-to-treat; SD = standard deviation.
